# What Matters in Piglets’ Exposure to Antibiotics Administered through Drinking Water?

**DOI:** 10.3390/antibiotics10091067

**Published:** 2021-09-03

**Authors:** Malika Chassan, Anne Hémonic, Didier Concordet

**Affiliations:** 1 Innovations Thérapeutiques et Résistances (INTHERES), Université de Toulouse, INRAe, ENVT, 31000 Toulouse, France; didier.concordet@envt.fr; 2 IFIP—French Pork and Pig Institute, 35650 Le Rheu, France; anne.hemonic@ifip.asso.fr

**Keywords:** pharmacokinetics, pharmacodynamics, exposure response, variability, antibiotics

## Abstract

A number of drugs are given in drinking water in piglet farming, although this way of administering drugs leads to significant and uncontrolled variability in exposures. Three main explanations for this variability have been described in the literature: (1) the drinking behavior of animals, (2) the drug concentration in water, and (3) the inter-individual variability in the pharmacokinetic (PK) parameters. This article assesses the relative importance of these three sources of exposure variability for doxycycline and amoxicillin using pharmacokinetic simulations and by observing watering behavior, and analyzes the consequences of this exposure variability. The water consumption behavior was by far the most important factor as it led to a variation in exposures of up to a factor of 7 between piglets. The second most influential factor was the drug concentration in the drinking water with variations ranging from −43.3% to +48.7% at the beginning and the end of the pipeline. Finally, the between-individual variation in PK parameters depends on the drug, but had a low impact on exposure variability. In the most variable case (doxycycline), the mean ratio between the 10% less exposed and the 10% most exposed piglets varied from 3.7 without PK parameters variability to 6 with PK variability. For both drugs, this study also showed that only a small percentage of the piglets (36%) could be considered as well exposed in case of infection by *Actinobacillus pleuropneumoniae* or *Pasteurella multocida*. There may be some existing technical ways to reduce this important variability. However, their cost and ease of implementation merit examination.

## 1. Introduction

Antimicrobial distribution through drinking water is widely used in commercial farming, especially for species for which individual treatment is not the best strategy. Indeed, collective treatment through drinking water allows metaphylaxis, that is, the treatment of a whole group of individuals when only a few animals show clinical signs of disease.

Even if this method of treatment appears simple to implement, it does lead to a large variation in the exposures. In a survey of 25 medium to large single-site and multi-site pig farming enterprises, Little et al. [1] demonstrated a wide variation in the choice and use of dosing equipment, the methods for calculating the dose and in preparing antibiotic stock solutions, the commencement time and duration of each daily dosing event, and the frequency of the administration of metaphylaxis.

Prats et al. [2] showed that doxycycline in water given ad libitum to 20–25 kg piglets for 5 days to led an average area under the plasma antimicrobial concentration–time curve over 24 h (AUC_24h) of 27.7 mg·h/L (SD: 24.8 mg·h/L).

Individually penned weaned piglets treated with amoxicillin in the drinking water (0.75 mg/mL) for a 4-h period on 2 consecutive days, exhibited a median AUC of 65 mg·h/L, ranging from 33.8 to 153.4 mg·h/L for a median (computed) dose of 26 mg/kg [3].

This huge variability in exposures implies that some animals are under-exposed while others are over-exposed. This variability in exposure could explain the clinical failure that occurs in some animals and facilitate the emergence of resistance [4]. It follows that understanding the source of this variability should enable the dosage regimens to be designed for more effective treatment, and also to minimize the development of antimicrobial resistance [5,6]

A recent review of pigs’ exposure to antibiotics distributed through their drinking water [7] identified three factors causing this variability:

1. The real concentration of drug present in the water drunk. Vandael et al. [8] showed that there was a large variation in the drug concentration at the end of the pipeline, and that it rarely reached the recommended therapeutic concentration range.
2. The watering behavior of animals, i.e., the variation in the dose actually ingested by each individual. Drinking behavior can be influenced by many factors such as diarrhea, temperature, and the water and food quality [9,10,11,12,13].
3. The difference in kinetic parameters between individuals, causing the animals to absorb and/or eliminate the antibiotic differently from each over.

Even if identified, their relative impact of these factors on pigs’ exposure and also on pathogens’ exposure still needs to be quantified.

In this work, simulations of plasma concentration profiles were obtained from real water consumption profiles to determine the relative influence of the pharmacokinetic (PK) parameters variability and of the watering behavior profile on the variation in exposures, specifically for two antibiotics widely used in piglet farming, amoxicillin and doxycycline. The expected efficacy of treatments on hypothetical infections by two common pathogens, *Actinobacillus pleuropneumoniae* and *Pasteurella multocida* were also investigated through the pharmacokinetic/pharmacodynamic (PK/PD) index.

## 2. Results

**Figure 1:**
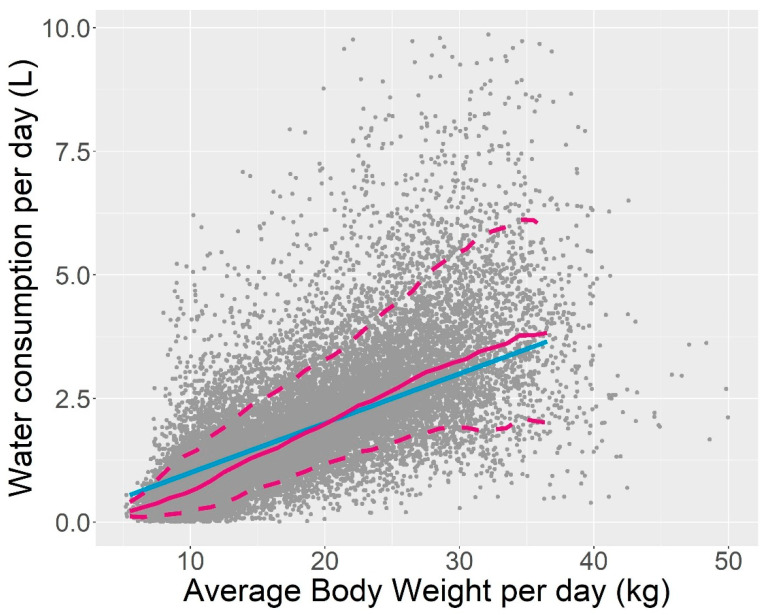
Daily water intake versus body weight for all the piglets and all the days. The blue lines correspond to 0.1 × body weight. The pink curves correspond to the 10%, 50% and 90% quantiles of water consumption for a given weight. For a given body weight, the daily water intake varies by more than 170%.

Figure 1 shows the daily water consumption against the average daily body weight for all data (a point corresponds to a given piglet for a given day). The middle line corresponds to the consumption estimated by 10% of the body weight. This rule of thumb is often used in practice to predict the water intake of pigs, based on a prediction of their weight for a given day [14,15]. This rough estimate tends to overestimate the water consumption for small piglets (less than 15 kg) and underestimate it for other animals. The upper and lower dashed curves correspond to the empirical quantiles at the level of 10% and 90% of the daily water intake for a given body weight, and the plain middle curve corresponds to the median. For a given weight, the water intake varies between the lower and upper quantiles by a factor of 2.7 to 7. These factors are presented in Table 1.

**Figure 2:**
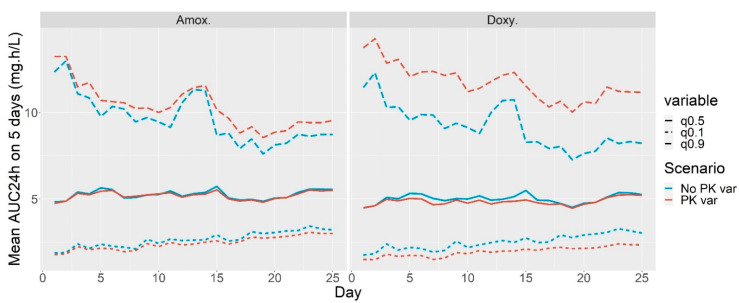
Median, 10% and 90% empirical quantiles of the AUC_24h distribution according to the day that the five-day treatment began, for amoxicillin (left) and doxycycline (right). The red curves correspond to simulations done with between-animal PK variability and the blue curves correspond to simulations done with the same PK parameters for all animals. The individual PK parameters variability has a very small influence on AUC_24h variability for amoxicillin. It has little impact on the 10% quantile of AUC_24h distribution for doxycycline and a larger impact on the 90% quantile.

Figure 2 shows the 10% and 90% quantiles and the median of the AUC_24h according to the first day of treatment for amoxicillin (top) and doxycycline (bottom). The red lines correspond to the simulations done for the first scenario, i.e., with PK variability. The blue lines correspond to the analogous quantiles for the second scenario, i.e., the same PK parameters for all the piglets. The dispersion seen between the red lines is the result of the variability in individual water consumption plus the individual pharmacokinetic variability. Conversely, the dispersion seen between the blue lines is due solely to the between-individual water consumption variability.

The higher values in the AUC_24h 90% quantile for the treatments beginning on days 1 and 2 are the consequence of some very high water consumption with respect to body weight during the very first days of post-weaning (this already observed fact [9] is not clearly visible in Figure 1 since the x-axis corresponds to the weight and not to the time, but is evident when the daily water intake by kilogram of body weight is plotted separately for each day—graphic not shown). The increase in the AUC_24h 90% quantile on days 13 and 14 is a consequence of the inclusion of one pen with very high water consumption (the average water consumption was 0.18 L/kg against 0.10 L/kg). Although high, this level of consumption is not aberrant, and there was no reason to exclude this pen from the study.

Apart from these variations in the 90% quantile, the distribution of AUC_24h is rather stable according to the first day of treatment.

Concerning the amoxicillin exposure, one can see that there is high variability in the AUC_24h, for example, for amoxicillin with PK variability, the inter-decile range (the difference between the 10% and 90% quantiles) is 7.72 with a median value of 5.20. The quantiles were computed considering all the AUC_24h values for all the individuals and all the first days of treatment, i.e., without considering each first day of treatment separately. The 10% most exposed piglets had a minimum AUC_24h more than 4 times larger than the maximum AUC_24h of the 10% least exposed piglets.

The variability in AUC_24h for amoxicillin is very similar in the simulations with PK parameters variability and without. One can see a very slight increase in the inter-decile range from 6.89 to 7.72 when the PK variability is included in the simulations. When the PK variability was excluded, the 10% most exposed piglets had a minimum AUC_24h 3.6 times larger than the maximum AUC_24h of the 10% least exposed piglets, versus 4 when the PK variability was included (Table 2).

Concerning the doxycycline exposure, as shown in the bottom graph in Figure 2, the addition of PK variability increases the inter-decile range from 6.69 to 9.82. This influence is especially noticeable in the 90% quantile, which is increased by 2.62 mg h/L on average. The ratio between the minimal AUC_24h of the 10% most exposed and maximum AUC_24h of the 10% least exposed piglets is 6.0 for the simulation with PK variability and 3.7 for the simulation without PK variability (Table 2).

**Figure 3:**
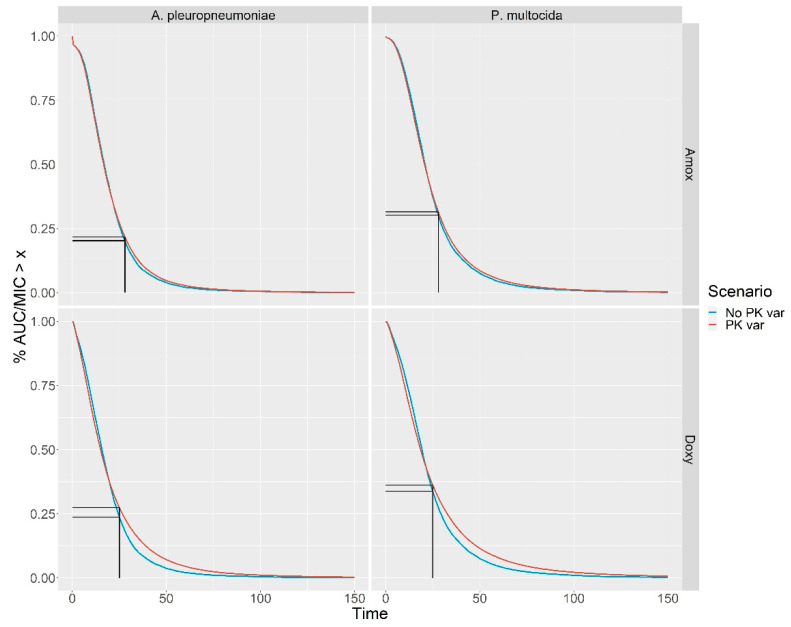
Survival function of the AUC/MIC ratio for amoxicillin (top) against *P. multocida* (left) and *A. pleuropneumoniae* (right) and for doxycycline (bottom) against *P. multocida* (left) and *A. pleuropneumoniae* (right). The vertical lines correspond to the threshold for bacteriostatic effect (28 h for amoxicillin and 25 h for doxycycline). At best, 36% of the piglets have an AUC/MIC ≥ 25 (doxycycline VS *P. multocida*).

The distributions of the AUC/MIC ratios are shown in Figure 3. For a given value x on the x-axis, the value on the y-axis corresponds to the percentage of piglets with an AUC/MIC ratio greater than x. The curves with and without individual PK variability are both drawn, even though they do not show significant differences. Only the simulations with PK variability are discussed. One can see a rapid decrease in the curves, which suggests there was insufficient exposure. For amoxicillin, the threshold ratio for a bacteriostatic effect has been evaluated at 28 h [16] for *P. multocida*. For *P. multocida* (top left graph), 31% of the piglets have an AUC/MIC ratio greater than 28 h. With regard to *A. pleuropneumoniae* (top right graph), only 21% of the piglets reach this bacteriostatic threshold. The doxycycline exposure is shown in the two bottom graphs of Figure 3. The bacteriostatic threshold was fixed at 25 h [17]. For *P. multocida* and *A. pleuropneumoniae*, 36% and 27%, respectively, of the piglets are sufficiently exposed.

In order to achieve the goal of, for example, 90% of individuals with an AUC/MIC ratio greater than the threshold ratio for bacteriostatic effect, as suggested in [5], the plasma concentrations of antibiotic should be multiplied by 3.5 for amoxicillin vs. *P. multocida* and by 5 for amoxicillin vs. *A. pleuropneumoniae*. For doxycycline, these concentrations should be increased by factors of 5.0 and 7.1, respectively. This implies that the concentration of antibiotics in drinking water should be also multiplied by the same factors.

## 3. Discussion

Drinking behavior has by far the greatest influence on drug exposure variability, with variations of up to 600% between piglets, for example, at the beginning of the post-weaning period. The highest water volumes in Figure 1 were deliberately not removed. There is no doubt that some of these volumes were not actually drunk by the piglets but rather they were lost by the piglets playing with the bowl drinker. The use of an anti-wastage push-lever on the bowl drinker limits this issue [10]. Nevertheless, this waste is inherent to the behavior of piglets and it is very difficult to differentiate between piglets that drink a lot and those that waste water, based only on the water consumption recorded at each visit to the drinking bowl. We can see in this figure that there is a continuum in the water consumption records. It would have been quite subjective to fix a limit, above which some of these volumes would have been discarded because they were not drunk. In this respect, the volume of water presented here overestimates the volume of water actually drunk. Anyway, discarding some of the results for high volume of water intake would not have been sufficient to reduce the variability in water intake for a given body weight. We chose to show only some quantiles of the water consumption distribution and AUC_24h distribution, which are robust to possible extreme values.

As shown in Figure 1, the rule of thumb that a piglet drinks approximately 10% of its body weight is reasonable and can be used as a first approximation. This rule of thumb overestimates, but by no more than 50%, the median water consumption up until a body weight of 15 kg and underestimates it after that. However, it cannot capture the large variability around this median.

The volume of water drunk was highly variable. As shown by the line q0.90/q0.10 in Table 1, for a given weight, the 10% heaviest drinkers drink at least 2.7 times more water than the 10% lightest drinkers, an increase of 170%. This factor increases when the weight decreases. For the lightest piglets (BW under 9.5 kg), this factor can reach a value of 7 (600%) because some piglets drank very little. This could be explained by the fact that some animals were distressed by the beginning of the post-weaning period. Whatever quartiles are chosen to evaluate this dispersion, this component of the variability is entirely linked to the piglet’s drinking behavior; thus, it is difficult to decrease by human intervention as long as the drug is given ad libitum with water.

The influence of the variability in the real concentration of the drug present in the water drunk (based on the recommended therapeutics concentration) was estimated previously in [8,18]. These estimations were compared to the influence of the drinking behavior variability estimated in this paper. Even though variations in the drug concentration do not affect exposure to the same extent as the drinking behavior, they are the second most influential factor in controlling exposure variability. The reasons why the water/drug concentrations can vary and are likely to decrease, are perfectly described in [7] and will not be discussed here.

Whatever the reason, variations in concentration affects all the animals in a pen/room/farm in the same way. In other words, if the drug concentration decreases by 20% in the drinking water, all the exposures will be decreased by 20%, and consequently the median exposure, and in a counter-intuitive manner, the dispersion about this median will be decreased in the same proportion. In an extreme situation where the water contains no drug, the exposures will all be equal to 0, without any dispersion. The decrease in concentration due to stability issues has been found to be less than 5% in 24 h for a large majority of products [18]. The concentration at the end of the water pipeline has been measured as between −43.3% and +48.7% of the concentration at the beginning [8]. Even in these extreme cases, the variability in exposure is, for example, reduced by 50%, i.e., going from a factor of 6 to a factor of 3. The average body weight and the average water intake by day used in Equation (3) and in the simulations were the actual average body weight and water intake computed from all piglets for the corresponding day. Obviously, these data cannot be known in practice because both the average water intake and the average BW are only available once the piglet has drunk, i.e., at the end of the day. In a sense, Equation (3) is a kind of best-case scenario.

At the end, the PK inter-individual variability did not seem to have a significant impact on the drug exposure variability. The influence of PK variability on the AUC_24h variability was larger in the simulations for doxycycline than in those for amoxicillin. This might be explained by the fact that the between-individual variability in PK parameters was higher for doxycycline than for amoxicillin. For amoxicillin, the quantiles seem to be slightly modified by the suppression of PK parameter variability with a variation of 7% (a decrease for the 90% quantile and an increase for the 10% one). However, there is still a factor of 3.6 (260%) between the AUC_24h of the 10% most exposed piglets and 10% less exposed ones.

Beside the large variability in watering behavior, this study highlights the presence of underexposed piglets due their very low water consumption. The exposure of the less exposed piglets was probably overestimated, since some of their water consumption was overestimated due to wastage. Considering the hypothesis regarding an infection by *P. multocida* or *A. pleuropneumoniae,* this underexposure was evident for the two antibiotics studied and for all the days on which treatment began. Figure 3 shows that at least 60% of the piglets were not sufficiently exposed. In the better case, doxycycline against *P. multocida*, the dose of doxycycline in the drinking water should be multiplied by 3.5 to achieve a goal of 90% of individuals being well-exposed.

Note as well, the very low impact of PK variability on the percentage of well-exposed piglets, as further proof of the low contribution of PK to the overall variability.

Usually, drug exposure is quantified by the AUC over 24 h when the equilibrium is reached. While the concept of equilibrium is particularly relevant for equally timed and spaced drug intake, it becomes fuzzier when the times of the drug intake is random. For this reason, we deliberately decided to evaluate the exposure using the average AUC_24h over the five days of treatments.

## 4. Conclusions

This study presents the relative importance of the main factors affecting variability in exposure to amoxicillin and doxycycline in piglets. Several methods could be explored to reduce this variability.

A possible solution to decrease the impact of drinking behavior on the exposure variability would be to fine tune the antibiotic concentration in water for each piglet. This requires (1) identifying the piglet just before it begins drinking, and (2) modulating the antibiotic concentration in water according to the quantity of water (and thus of antibiotics) already drunk. Of course, it is difficult to predict exactly when a piglet is going to drink and how much water it will drink. However, if at the first drinking occasion of the day, the drug concentration in water is very high, it would remain to adjust approximately the drug concentration to reach approximately the target dose of drug per day. Proceeding so would certainly not completely prevent the dispersion of exposures due to the difference in water intake, but it would decrease it. Excellent solubility of the drug would also be needed because of the high concentration in the water.

Since the inter-individual PK variability does not really appear to be the main problem in the control of drug exposures, choosing a formulation with good PK properties could help to reduce the influence of the time and the amount of drug intake. As it is done for sustained-release forms, a formulation with a different bioavailability (a small Ka) should help to obtain the so-called “flip-flop” that is well-known by pharmacokineticists. Slowing down the input rate in this way should help to reduce, at least partially, the random times and doses drunk at each drinking occasion. However, it is questionable as to whether solubility problems can be resolved given such slow input rate formulations.

## 5. Materials and Methods

The water consumption and weights of 918 piglets were recorded during their post-weaning period. This period corresponds to ages ranging from 28 to 70 days. The 918 piglets originated from 9 consecutive batches and were studied from September 2017 to April 2019 (all-in/all-out management with 6 weeks of post-weaning plus 3 weeks of emptying). Each batch consisted of 102 piglets divided into 6 pens of 17 animals. Pens 1 to 3 were in the first room and pens 4 to 6 were in a second room. The rooms were separated by a door. The six pens were subject to the same farming conditions: the climatic environment was the same (temperature and ventilation controlled), the light was turned on and off at the same times (9 a.m. and 5 p.m.). Because of electronic device failures, the water consumption and weights were available only for some piglets on each day of the post-weaning period. Table 3 presents the number of piglets with complete water and weight recording for each day of the study. In addition, the simulations performed required a period of five consecutive days of data. Thus, for days 1 to 25, only the piglets for which the data were available over the 5 following days were used. Table 4 shows the number of piglets available for each day of treatment after the simulation began.

Each pen was equipped with two connected feeders and one connected water dispenser (bowl drinker). Food and water were delivered ad libitum. The water dispenser was also equipped with a connected weighing station. Each piglet was equipped with a radio frequency identification (RFID) ear tag. Each time an animal entered the weighing station/water dispenser, four pieces of data were recorded: the pig number, its water consumption (mL), its weight (kg) and the time of entry (in minutes from the beginning of post-weaning). The batch and pen identifiers were also saved.

The water consumption as a function of body weight is represented in Figure 1. The 10%, 50% and 90% water consumption percentiles appearing in this figure were computed using a regression quantile method as implemented in the quantreg package of the R software [19].

The time-profile for the water consumption of each piglet was used to simulate its exposure to two antibiotics using two scenarios. In the first scenario, the individual PK parameters varied among piglets while in the second scenario considered, the PK parameters were the same for all piglets. Next, a comparison of the exposures obtained with the two scenarios allowed us to evaluate the weight of varying kinetics parameters.

The animals’ exposure to amoxicillin and doxycycline, two oral antibiotics frequently used in pig farming [2,20] was studied. The population pharmacokinetics of these have already been described for doxycyxline [21] and for amoxycillin [22]. For both antibiotics, a two-compartmental model best described the time course of their plasma concentrations, namely, for a single dose *D* of drug, its time course was described by

(1) $$Z_{t}=Df\left(t,\psi \right)+\sigma Df\left(t,\psi \right)\varepsilon_{t}$$

where $Z_{t}$ is the drug plasma concentration at time t after its administration orally, ψ is the vector containing the individual PK parameters for the drug. In the first scenario, these parameters were free to vary randomly from one individual to another. As in [21,22], we assumed that ψ was distributed according to a log-normal distribution whose parameters. The parameters for amoxicillin were extracted from Tables 1 and 2 of [22] and those for doxycycline were extracted from Table 1 of [21]. In the second scenario, the value of ψ was fixed for all piglets to the median values.

The function f(t,ψ) is the expected plasma concentration at time t for a dose D=1  of drug for a two-compartmental model with extravascular entry, σ is the coefficient of variation of $Z_{t}$ and finally, the ${\left(\varepsilon_{t}\right)}_{t}\;$ are independent N(0,1) random variables.

Notice that because the function f gives the expected concentration for an extravascular route, f(0,ψ)=0.

It remains to describe the model for several administrations. Assume that a piglet drank at times ${\left(T_{k}\right)}_{k=1,\ldots ,K}$ the doses ${\left(D_{k}\right)}_{k=1,\ldots ,K}$ of drug, then the plasma concentration at time t is given by

(2) $$Y_{t}=\overset{K}{\underset{k=1}{\sum }}D_{k}f\left({\left(t-T_{k}\right)}_{+},\psi \right)+\sigma \overset{K}{\underset{k=1}{\sum }}D_{k}f\left({\left(t-T_{k}\right)}_{+},\psi \right)\varepsilon_{t}$$

where ${\left(t-T_{k}\right)}_{+}=t-T_{k}$ when $t>T_{k}$ and ${\left(t-T_{k}\right)}_{+}=0$ otherwise.

The dose $D_{k}$ is directly proportional to the volume $V_{k}$ of water drunk by the piglet and to the drug concentration in water $C_{day\left(k\right)}$ chosen for the day when the piglet drank, more precisely, $D_{k}=V_{k}\times C_{day\left(k\right)}$.

For all simulations, the concentration of antibiotic in the drinking water was chosen to comply with the recommendations: an average daily dose (ADD) of 20 mg/kg for amoxicillin and 10 mg/kg for doxycycline. For the simulations, the concentration in drinking water was fixed each day to:

(3) $$C_{day}=ADD\times \;\frac{Average\;body\;weight\;day}{Average\;water\;intake\;day}$$

The average body weight and the average water intake by day are the actual average body weight and water intake computed for all piglets for the corresponding day. The simulations assumed that the drug concentration in the water changed each day and always met the therapeutic recommendations.

Five-day treatments were simulated, with the first day of treatment varying from day 1 to 25. For each pig (i.e., each water consumption profile), ten samples of kinetic parameters were drawn from the distributions depicted in Table 3, and then, ten concentration profiles were computed.

The drug exposure was evaluated by the area under the concentration curve (AUC) over 24 h starting at 0 h to 24 h of the day. The AUC over 24 h was denoted as AUC_24h. Because the AUCs obtained for each day of treatment were not the same, an average AUC was computed over the five days of treatment. The influence of the day that the treatment began was also investigated by changing the first day of treatment from day 1 to day 25.

The comparison of the AUC distributions obtained for the two scenarios allowed us to quantify the relative impact of the PK between-subject variability on exposures’ variability. For these comparisons to be meaningful, the distributions were based on the ratio between quantiles 90% and 10% of the AUCs obtained for the two scenarios. Empirical quantiles were used. They were computed for each day when indicated and for the entire period when not specified.

As suggested by many authors, the clinical consequences of this exposure variability can be roughly evaluated by the PK–PD index from AUC/MIC (in hours) to predicts efficacy [16]. We illustrated the possible use of these exposure distributions by using *P. multocida* and *A. pleuropneumoniae* whose MIC distributions are taken from the EUCAST website [23] for doxycycline and from [24] for amoxicillin (Table 4a for *P. multocida* and Table 4b for *A. pleuropneumoniae*).

For a given bacteria, when the AUC/MIC index is high, the individual is considered to be well exposed. Thus, for each couple (bacteria, antibiotic) the curves for the percentage of individuals with an AUC/MIC greater than x (P(AUC/MIC≥x)) versus x were represented.

The functions P(AUC/MIC≥x) were calculated using a conditioning/deconditioning argument, namely:

(4) $$P\left(\frac{AUC}{MIC}\geq x\right)=P\left(AUC\geq xMIC\right)=\underset{mic}{\sum }P\left(AUC\geq x\;mic\right)P\left(MIC=\;mic\right)$$

The values of P(MIC=mic) are given in Table 4. Equation (4) shows that to deduce P(AUC/MIC≥x), it suffices to combine the values of P(AUC≥x mic) for each value of mic, that is, the percentage of individuals with an AUC greater than (x mic).

## Figures and Tables

**Table 1:** Daily water intakes (in liters) according to weight. There is a factor of at least 2.7 (170%) between the volume of water drunk by the 10% heaviest drinkers and the 10% lightest drinkers. This factor reaches 7 (600%) at the beginning of the post-weaning period.

Body weight (kg)	6.5	9.5	12.5	15.5	18.5	21.5	24.5	27.5	30.5	33.5
q0.1 (L)	0.10	0.13	0.16	0.19	0.26	0.30	0.39	0.50	0.65	0.72
Median (L)	0.30	0.38	0.49	0.57	0.68	0.83	1.01	1.15	1.29	1.40
q0.9 (L)	0.58	0.85	1.11	1.33	1.46	1.73	1.91	2.14	2.36	2.50
q0.9/q0.1	5.72	7.00	4.91	3.47	3.00	2.72	2.74	2.78	2.89	3.14

**Table 2:** Quantile of AUC_24h distribution (in mg h/L) computed with and without variability in individual PK parameters and considering all the AUC_24h values for all the individuals and all the first day of treatment. The PK variability has little impact on exposure for the less exposed animals, and a “medium” impact for the 10% most exposed animals.

	Amoxicillin		Doxycycline	
	with PK	without PK	with PK	without PK
Median	5.20	5.28	4.89	5.02
q0.1	2.46	2.63	1.95	2.46
q0.9	10.18	9.52	11.77	9.15
q0.9/q0.1	4.1	3.6	6.0	3.7

**Table 3:** Number of piglets with complete data recording on each day of the study.

Day	1	2	3	4	5	6	7	8	9
Nb. piglets	337	694	706	739	620	626	723	610	473
Day	10	11	12	13	14	15	16	17	18
Nb. piglets	541	440	538	405	204	356	406	608	541
Day	19	20	21	22	23	24	25	26	27
Nb. piglets	440	405	403	571	419	500	500	754	720
Day	28	29	30						
Nb. piglets	786	585	504						

**Table 4:** Number of piglets with complete data recording during the 5 days following the day given in the first row of the table. As an example, the complete water consumption and body weights were available for 230 piglets on day 1, 2, 3, 4, 5 after the beginning of the post-weaning period.

Day	1	2	3	4	5	6	7	8	9
Nb. piglets	230	229	508	459	286	286	221	237	170
Day	10	11	12	13	14	15	16	17	18
Nb. piglets	102	102	201	201	201	102	170	204	253
Day	19	20	21	22	23	24	25		
Nb. piglets	187	268	268	234	320	337	318		

## Data Availability

The data presented in this study are available on request from the corresponding author. The data are not publicly available due to a confidentiality clause.

## References

[1] Little S., Woodward A., Browning G., Billman-Jacobe H. (2021). In-Water Antibiotic Dosing Practices on Pig Farms. Antibiotics.

[2] Prats C., El Korchi G., Giralt M., Cristòfol C., Peña J., Zorrilla I., Saborit J., Pérez B. (2005). PK and PK/PD of Doxycycline in Drinking Water after Therapeutic Use in Pigs. J. Vet. Pharmacol. Ther..

[3] Jensen G.M., Lykkesfeldt J., Frydendahl K., Møller K., Svendsen O. (2006). Pharmacokinetics of Amoxicillin Administered in Drinking Water to Recently Weaned 3- to 4-Week-Old Pigs with Diarrhea Experimentally Induced by Escherichia Coli O149:F4. Am. J. Vet. Res..

[4] Ferran A.A., Roques B.B. (2019). Can Oral Group Medication Be Improved to Reduce Antimicrobial Use?. Vet. Rec..

[5] Toutain P.L., Lees P. (2006). WS04 The Population PK/PD Approach for a Rational Use of Anti-Infective Drugs to Minimize Resistance. J. Vet. Pharmacol. Ther..

[6] Bon C., Toutain P.L., Concordet D., Gehring R., Martin-Jimenez T., Smith J., Pelligand L., Martinez M., Whittem T., Riviere J.E. (2018). Mathematical Modeling and Simulation in Animal Health. Part III: Using Nonlinear Mixed-Effects to Characterize and Quantify Variability in Drug Pharmacokinetics. J. Vet. Pharmacol. Ther..

[7] Little S.B., Crabb H.K., Woodward A.P., Browning G.F., Billman-Jacobe H. (2019). Water Medication of Growing Pigs: Sources of between-Animal Variability in Systemic Exposure to Antimicrobials. Animal.

[8] Vandael F., de Carvalho Ferreira H.C., Devreese M., Dewulf J., Daeseleire E., Eeckhout M., Croubels S. (2020). Stability, Homogeneity and Carry-Over of Amoxicillin, Doxycycline, Florfenicol and Flubendazole in Medicated Feed and Drinking Water on 24 Pig Farms. Antibiotics.

[9] Fraser D., Patience J.F., Phillips P.A., McLeese J.M. (1990). Water for Piglets and Lactating Sows: Quantity, Quality and Quandaries. Recent Adv. Anim. Nutr..

[10] Torrey S., Toth Tamminga E.L.M., Widowski T.M. (2008). Effect of Drinker Type on Water Intake and Waste in Newly Weaned Piglets. J. Anim. Sci..

[11] Vermeer H.M., Kuijken N., Spoolder H.A.M. (2009). Motivation for Additional Water Use of Growing-Finishing Pigs. Livest. Sci..

[12] Patience J.F. (2012). The Importance of Water in Pork Production. Anim. Front..

[13] Ahmed S.T., Mun H.-S., Yoe H., Yang C.-J. (2015). Monitoring of Behavior Using a Video-Recording System for Recognition of Salmonella Infection in Experimentally Infected Growing Pigs. Animal.

[14] Massabie P., Aubert C., Ménard J.L., Roy H., Boulestreau-Boulay A.L., Dubois A., Dezat E., Dennery G., Roussel P., Martineau C. (2013). Maîtrise Des Consommations d’eau En Élevage: Élaboration d’un Référentiel, Identification Des Moyens de Réduction, Construction d’une Démarche de Diagnostic. Innovations Agronomiques. Innov. Agron..

[15] Rousselière Y., Hémonic A., Marcon M. Monitoring of the Individual Drinking Behavior of Healthy Weaned Piglets and Pregnant Sows. Proceedings of the The 8th European Conference on Precision Livestock Farming.

[16] Lees P., Pelligand L., Illambas J., Potter T., Lacroix M., Rycroft A., Toutain P.-L. (2015). Pharmacokinetic/Pharmacodynamic Integration and Modelling of Amoxicillin for the Calf Pathogens Mannheimia Haemolytica and Pasteurella Multocida. J. Vet. Pharmacol. Ther..

[17] Andes D., Craig W.A., Nightingale C.H., Ambrose P.G., Drusano G.L., Murakawa T. (2007). Pharmacokinetics and Pharmacodynamics of Tetracyclines. Theory and Clinical Practice.

[18] Boeren M., Michiels D., Verhoeve P., van Aken K., de Keijser H. Amoxicillin, Stability and Solubility. Proceedings of the World Poultry Science Association (WPSA) XII European Poultry Conference.

[19] Quantreg: Quantile Regression. https://cran.r-project.org/web/packages/quantreg/quantreg.pdf.

[20] Godoy C., Castells G., Martí G., Capece B.P.S., Pérez F., Colom H., Cristòfol C. (2011). Influence of a Pig Respiratory Disease on the Pharmacokinetic Behaviour of Amoxicillin after Oral Ad Libitum Administration in Medicated Feed. J. Vet. Pharmacol. Ther..

[21] del Castillo J.R.E., Laroute V., Pommier P., Zemirline C., Keita A., Concordet D., Toutain P.-L. (2006). Interindividual Variability in Plasma Concentrations after Systemic Exposure of Swine to Dietary Doxycycline Supplied with and without Paracetamol: A Population Pharmacokinetic Approach1. J. Anim. Sci..

[22] Rey J.F., Laffont C.M., Croubels S., De Backer P., Zemirline C., Bousquet E., Guyonnet J., Ferran A.A., Bousquet-Melou A., Toutain P.-L. (2014). Use of Monte Carlo Simulation to Determine Pharmacodynamic Cutoffs of Amoxicillin to Establish a Breakpoint for Antimicrobial Susceptibility Testing in Pigs. Am. J. Vet. Res..

[23] EUCAST. https://mic.eucast.org/search/.

[24] El Garch F., de Jong A., Simjee S., Moyaert H., Klein U., Ludwig C., Marion H., Haag-Diergarten S., Richard-Mazet A., Thomas V. (2016). Monitoring of Antimicrobial Susceptibility of Respiratory Tract Pathogens Isolated from Diseased Cattle and Pigs across Europe, 2009–2012: VetPath Results. Vet. Microbiol..

